# Integrated Metabolomic and Transcriptomic Analysis Revealed the Mechanism of BHPF Exposure in Endometrium

**DOI:** 10.3390/toxics13020100

**Published:** 2025-01-27

**Authors:** Xin Tan, Nengyong Ouyang, Wenjun Wang, Junting Qiu

**Affiliations:** 1 Reproductive Medicine Center, Department of Obstetrics and Gynaecology, Sun Yat-sen Memorial Hospital, Sun Yat-sen University, Guangzhou 510120, China; tanx27@mail.sysu.edu.cn (X.T.); ouyny@mail.sysu.edu.cn (N.O.); 2 Guangdong Provincial Key Laboratory of Malignant Tumor Epigenetics and Gene Regulation, Sun Yat-sen Memorial Hospital, Guangzhou 510120, China; 3 State Key Laboratory of Organic Geochemistry, Guangzhou Institute of Geochemistry, Chinese Academy of Sciences, Guangzhou 510640, China

**Keywords:** fluorene-9-bisphenol (BHPF), endometrium, transcriptome, metabolome, mechanism

## Abstract

Fluorene-9-bisphenol (BHPF) has been increasingly used as a bisphenol A substitute in the synthesis of various products. Previous studies have suggested that BHPF can be released from plastic bottles into drinking water, and BHPF accumulation has been reported to cause various adverse effects in humans. Nevertheless, the impact of BHPF exposure on endometrial epithelial cells remains largely unexplored. Here, we investigated the effects of exposure to different concentrations of BHPF on endometrial cells and used integrated metabolomic and transcriptomic methods to elucidate the underlying molecular mechanisms. Our results revealed significant associations between specific metabolites and genes, indicating that low-concentration exposure to BHPF affects endometrial epithelial cells by targeting pathways related to primary immunodeficiency, in which the key genes are *IL7R* and *PTPRC*. High-concentration exposure to BHPF decreased cell viability by regulating the purine metabolism pathway, as well as dysregulating the expression of *PGM1*, *PDE3B*, *AK9,* and *ENTPD8*. Our study highlights that the health risk of BHPF exposure to endometrial epithelial cells is concentration-dependent and that integrated analysis of metabolomic and transcriptomic data not only revealed the biological effects of BHPF and its underlying mechanisms, but also provided key candidate target genes for further exploration.

## 1. Introduction

Fluorene-9-bisphenol (BHPF), also referred to as 9,9-bis(4-hydroxyphenyl)-fluorene, is extensively utilized in the production of a diverse range of products, including protective coatings, composites, molded products, and insulation materials [1,2,3]. In recent years, as a substitute for bisphenol A (BPA), BHPF has been a rising application in the manufacturing of food contact materials and containers, such as drinking bottles, baby bottles, and feeding bottles, in response to the restrictions or bans imposed on BPA use [4]. Notably, BHPF has been detected in the serum of individuals who have consumed water out of beverage bottles made with BHPF over an extended period of time, suggesting that BHPF can be released from plastic bottles into drinking water and accumulate in humans [4]. Moreover, the concentrations of BHPF were recorded to reach 0.069 and 0.067 ng/L in the Hunhe River and Liaohe River, respectively, in Northeast China [5]. According to field investigations conducted in Beijing, BHPF was detected in 60% of surface waters, with a mean value of 10.49 ± 6.33 ng/L [6]. Zhang et al. reported that even at exposure concentrations below the no-observed-adverse-effect-level (NOAEL) of BPA, BHPF can still interfere with endocrine function and produce antiestrogenic effects in mice [4], indicating that BHPF potentially has a stronger endocrine-disrupting adverse effect on the endocrine system than BPA. In addition, recent studies revealed that short-term exposure of zebrafish (*Danio rerio*) to high BHPF levels causes thyroid disruption and histopathological changes in gill and liver tissues [7], and disrupts myelination by influencing the hypothalamic-pituitary-thyroid axis [8]. In addition, acute exposure to BHPF led to early developmental abnormalities, delayed cardiac morphogenesis, and impaired cardiac contractility of zebrafish [9]. Adverse impacts on behavior, locomotor activity, development, and lipid metabolism were also observed in embryonic or larval zebrafish after exposure to BHPF [10,11]. Moreover, experimental studies in mice have shown that BHPF can injure the liver and ovarium, inhibit the maturation of Leydig cells, and induce anxiety- and depression-like behaviors [12,13,14,15,16]. Additionally, BHPF treatment induced inflammation and ulcers in the colon and rectum in a mouse model of colitis [17]. High levels of BHPF were also shown to suppress the synthesis of aldosterone, cortisol, testosterone, and estradiol by downregulating steroidogenic genes in H295R cells via the AC/cAMP/PKA signaling pathway [18]. These studies indicate that BHPF is a threat to the health and safety of animals that should not be neglected.

On the other hand, numerous studies have been undertaken to investigate the effects of BHPF on the reproductive system. Jia et al. demonstrated that 100 μM BHPF exposure for 14 h could induce cytotoxicity in mouse oocytes and cause ovarian damage [13]. Jiao et al. reported that treatment with 100 μM BHPF for 12 h could affect the meiosis maturation of mouse and porcine oocytes by inhibiting the emission of the first polar body [19]. Another study suggested that 50 μM BHPF induced oxidative stress, decreased the mitochondrial membrane potential, and caused DNA damage in porcine Sertoli cells after 48 h of exposure [20]. In addition, research on placental cells has shown that 5 μg/mL BHPF affects cell viability and necrosis [21]. Moreover, recent studies demonstrated the significance of BHPF exposure on endometrial cells. Wang et al. reported that BHPF inhibits the epithelial–mesenchymal transition of Ishikawa cells by repressing the TGF-β signaling pathway [22]. Jin et al. performed an in vivo study and reported that 10 μM BHPF inhibits decidualization in both mouse and human models during early pregnancy [23]. Nevertheless, the potential effects of BHPF exposure on human endometrial epithelial cells and the underlying mechanisms remain largely unexplored.

In this study, we performed in vitro assays using Ishikawa cells, a commonly employed model for investigating endometrial physiology and pathology, to obtain new insights at the metabolomic and transcriptomic levels.

## 2. Materials and Methods

### 2.1. Chemicals and Reagents

Fluorene-9-bisphenol (BHPF, ≥98%, HPLC grade) was acquired from Yuanye Bio-Technology Co., Ltd. (Shanghai, China). Dimethyl sulfoxide (DMSO) (>99%, GC grade) was obtained from Sigma-Aldrich (St. Louis, MO, USA). Methanol (≥99.9%, HPLC grade) was procured from Aladdin Biochemical Technology Co., Ltd. (Shanghai, China). Phosphate-buffered saline (PBS) was sourced from Thermo Fisher Scientific (Carlsbad, CA, USA). TRIzol reagent was obtained from Sigma–Aldrich Trading Co., Ltd. (Shanghai, China).

### 2.2. Cell Culture and Exposure to Chemicals

Human endometrial adenocarcinoma Ishikawa cells were purchased from Guangzhou Cellcook Biotech Co, Ltd. (Guangzhou, China). Ishikawa cells were maintained in RPMI 1640 (GIBCO, Grand Island, NY, USA) enriched with 15% (*v*/*v*) fetal bovine serum (FBS, Shanghai Excell Biological Technology Co., Ltd.), and 1% (*v*/*v*) penicillin–streptomycin (Gibco, Grand Island, NY, USA). The cells were incubated at 37 °C in a humidified incubator with 5% CO_2_.

During the in vitro assays, BHPF was initially dissolved in DMSO and subsequently diluted with medium to prepare a series of working concentrations (0, 2.5, 3.75, 5, 6.25, 12.5, 25, 37.5, 50, 62.5, 75, 87.5, and 100 μM) with final DMSO concentrations of ≤0.02% (*v*/*v*). The scramble group was cultured under the identical culture conditions, absent of both BHPF and DMSO, and the control group was maintained under equivalent conditions with 0.02% DMSO (*v*/*v*). The effects of BHPF were analyzed after 48 h of exposure.

### 2.3. Cell Viability Assay

Ishikawa cells were plated at a density of 1 × 10^3^ cells per well in 96-well plates and incubated at 37 °C with 5% CO_2_. After cultured for 24 h, the culture medium was exchanged for fresh medium containing various working concentrations of BHPF, and the mixture was continuously incubated for 48 h. After incubation, the cells were washed with PBS and cell viability was assessed using the CCK-8 assay with a Cell Counting Kit-8 (Dojindo Molecular Technologies, Inc., Kumamoto, Japan) following the manufacturer’s instructions. All values represent the mean of three independent experiments conducted in triplicate.

### 2.4. Cell Treatment for Metabolomics and Transcriptomics

A total of 5 × 10^6^ cells were initially plated in 10 cm culture dishes. When 60% confluency was reached after 24 h of culture, the cells were exposed to varying concentrations of BHPF (0.5 μM or 5 μM). Medium containing 0.02% DMSO was used as a vehicle control. After treatment with 0.5 μM or 5 μM of BHPF or 0.02% DMSO for 48 h, the cells were harvested for the following experiments. Three duplicates were prepared for each group. For the metabolomics study, culture medium was carefully aspirated from each plate, and the cells were washed with ice-cold PBS twice to remove residual medium. The cells were quenched with 1.5 mL of ice-cold methanol and then collected with cell scrapers. The cell samples were subsequently centrifuged at 800 rpm for 10 min at 4 °C. The supernatant was evaporated, and the cells were snap frozen in liquid nitrogen. The samples were subsequently stored at −80 °C before metabolite extraction and metabolomics analysis. For transcriptome profiling, the cells were washed with sterile PBS before the addition of 1 mL of TRIzol reagent for total RNA extraction after exposure to BHPF or 0.02% DMSO. The samples were stored at −80 °C before transcriptome sequencing.

### 2.5. Quantitative Reverse Transcription PCR

Total RNA was isolated using the RNAprep Pure Cell Kit (TIANGEN, Beijing, China) and was reverse-transcribed using M-MLV reverse transcriptase (Promega, Madison, WI, USA). cDNA was used as a template for quantitative reverse transcription PCR (qRT-PCR) using the Stratagene MX3000P Sequence Detection System with SYBR Green qPCR mixture (Invitrogen, Carlsbad, CA, USA). Each qRT-PCR experiment was performed in triplicate. ACTB was used as a loading control. The primer sequences of the indicated genes are provided in Supplementary Table S1.

### 2.6. Statistical Analyses

#### 2.6.1. Metabolomics Data Analysis

In this study, 18,714 peaks were detected and 380 metabolites remained after using a relative standard deviation denoising method. Missing values were imputed with half of the minimum observed value, and a normalization procedure was applied during data analysis. The resulting dataset, featuring the peak number, sample name, and normalized peak areas, was then imported into the SIMCA16.0.2 software (Sartorius Stedim Data Analytics AB, Umea, Sweden) for multivariate analysis. The data were scaled and subjected to logarithmic transformation to diminish the influence of noise and address the high variance among the variables. After transformations, principal component analysis (PCA) and orthogonal projections to latent structures–discriminant analysis (OPLS-DA) were performed. *R*^2^ and *Q*^2^ values were computed, and the variable importance in projection (VIP) values for the first principal component of the OPLS-DA model were determined. Metabolites exhibiting VIP values greater than 1 and *p*-values less than 0.05 (Student’s *t*-test) were deemed significantly altered. Pathway enrichment analysis was performed using commercial databases such as the Kyoto Encyclopedia of Genes and Genomes (KEGG) (http://www.genome.jp/kegg/ (accessed on date 21 January 2025)) and MetaboAnalyst (http://www.metaboanalyst.ca/ (accessed on date 21 January 2025)).

#### 2.6.2. Transcriptomic Data Analysis

Total RNA was isolated using TRIzol reagent (Thermo Fisher Scientific, Waltham, MA, USA) according to the manufacturer’s guidelines. RNA quantity and purity were evaluated with a Bioanalyzer 2100 and RNA 6000 Nano LabChip Kit (Agilent Technologies, Santa Clara, CA, USA), selecting high-quality RNA samples with a RIN value exceeding 7.0 for library preparation. The cDNA libraries exhibited an average insert size of 300 ± 50 bp. Paired-end sequencing (2 × 150 bp, PE150) was conducted on an Illumina NovaSeq™ 6000, following the recommended protocol of the manufacturer.

The raw sequencing data were initially processed with Trimmomatic (v 0.36) to generate clean reads. These data were aligned to the reference genome using HISAT2 (v 2.2.1.0). Gene expression levels were quantified by calculating fragments per kilobase per million (FPKM) values with Cufflinks, and read counts were determined using HTSeq-count (v 0.9.1). Differential expression analysis was carried out in R, with genes exhibiting a *p*-value < 0.05 and a fold change > 2 classified as differentially expressed genes (DEGs). Hierarchical clustering of DEGs was performed to elucidate gene expression patterns among various samples. Additionally, Gene Ontology (GO) and KEGG pathway enrichment analyses were conducted to further characterize the DEGs.

#### 2.6.3. Joint Analysis

Pathway-based joint analysis of all differentially expressed metabolites (DEMs) (*n* = 48) and DEGs (*n* = 29) was performed using MetaboAnalyst (v 5.0), with a *p*-value < 0.05 from the hypergeometric test. For correlation-based joint analysis, involving the representative DEMs (*n* = 6), DEGs (*n* = 6), and cytokines (*n* = 3), MetScape (v 3.8.2) was utilized. Spearman’s correlation coefficients (two-tailed) were applied to assess the nonparametric relationships. Additional data analyses were executed with GraphPad Prism (v 9.1.1). Statistical significance between control and experimental groups was evaluated using a one-way or two-way analysis of variance (ANOVA), with Bonferroni and Dunnett tests applied for post hoc comparisons following one and two-way ANOVA, respectively. A *p*-value < 0.05 was considered statistically significant.

## 3. Results

### 3.1. Effects of BHPF on Ishikawa Cell Viability

**Figure 1:**
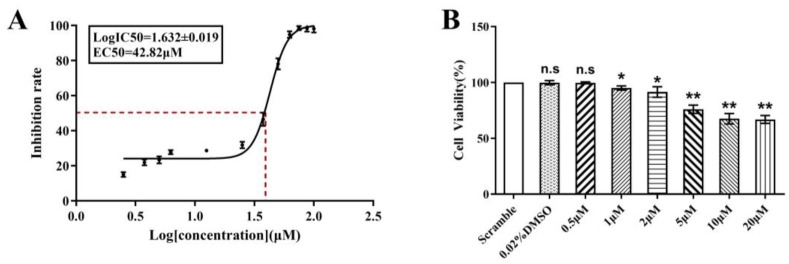
BHPF inhibits the cell viability of endometrial cells. (**A**) Viability curve at 48 h post-treatment, reflecting the percentage of viable cells across a BHPF gradient. (**B**) Cell viability of Ishikawa cells after treatment for 48 h was measured by CCK-8 assays. Data are presented as the mean ± SD of at least 3 independent experiments. * *p* < 0.05, ** *p* < 0.01. Abbreviation: EC50, median effect concentration; SD, standard deviation; n.s, not significant.

To assess whether human endometrial epithelial cells are affected by BHPF, we utilized the Ishikawa cell line as a study model for our research. Ishikawa cells are derived from an individual with endometrial adenocarcinoma and are a well-differentiated cell line with numerous advantages as a study model, providing a comprehensive representation of the state of endometrial cells. We first commenced the investigation by evaluating the impact of BHPF on the viability of Ishikawa cells through a CCK-8 assay. Ishikawa cells were exposed to BHPF at progressively higher concentrations (0, 2.5, 3.75, 5, 6.25, 12.5, 25, 37.5, 50, 62.5, 75, 87.5, and 100 μM) following 48 h of exposure, and the half-maximal inhibitory concentration (EC50) value was determined. As illustrated in Figure 1A, BHPF exhibited a concentration-dependent inhibition of Ishikawa cell proliferation, with a mean EC50 of 42.82 μM.

We subsequently conducted experiments with a series of low-concentration gradients to further investigate the impact of BHPF. Ishikawa cell survival remained largely unchanged at BHPF concentrations of 0.5, 1, or 2 μM when compared to scramble and control treatments, whereas cells exposed to 5 μM BHPF represented considerably increased cytotoxicity (*p* < 0.05) (Figure 1B). Therefore, the threshold concentration at which there was no significant reduction in cell viability or cell phenotype (0.5 μM) was chosen as the low concentration, and the lowest concentration at which cell viability was significantly inhibited (5 μM) was chosen as the high concentration for further studies. Notably, according to the data reported in the literature [23], the concentrations used for acute exposure research were significantly greater than those used for in vivo study; therefore, the concentrations we used in this study were rationally chosen and more closely related to the physiological conditions.

### 3.2. Metabolomic Analysis

#### 3.2.1. Effects of BHPF Exposure on Metabolic Patterns in Ishikawa Cells

To characterize the metabolic profile of the BHPF-exposed endometrial epithelial cells, nontargeted metabolomics (LC-MS/MS) analyses of cell extracts was carried out. A total of 18,714 peaks were identified, and 380 metabolites were screened. These metabolites were further categorized into 230 lipids and lipid-like molecules, 62 organic acids and derivatives, 34 organoheterocyclic compounds, 15 organic nitrogen compounds, and 14 benzenoids (Figure S1A), etc. PCA and OPLS-DA plots indicated that exposure of 0.5 μM and 5 μM BHPF resulted in metabolic profiles that were distinct from those of the control (Figure S2A,B).

By integrating VIP values > 1.0 with *p*-values < 0.5, 178 DEMs were found. All of the DEMs were visualized via a heatmap generated by hierarchical clustering analysis, and this visualization clearly grouped all biological replicates of the metabolomics samples together, reflecting the high repeatability and suitable homogeneity among the replicates (Figure S1B).

To further elucidate the metabolic changes that occurred during the accumulation of BHPF at 0.5 μM and 5 μM, all 178 annotated DEMs were sorted into nine clusters on the basis of their accumulation patterns via the K-means clustering algorithm (Figure S1C and Table S2). Using the accumulation patterns of the clusters, we discerned metabolites that exhibited selective enrichment in endometrial epithelial cells at different concentrations. The metabolites in cluster 3 and cluster 6 decreased gradually during the accumulation of BHPF, whereas those in clusters 5 and 7 significantly accumulated during the accumulation process, suggesting that they are closely related to the cytotoxicity of BHPF.

#### 3.2.2. Differentially Expressed Metabolite Identification

**Figure 2:**
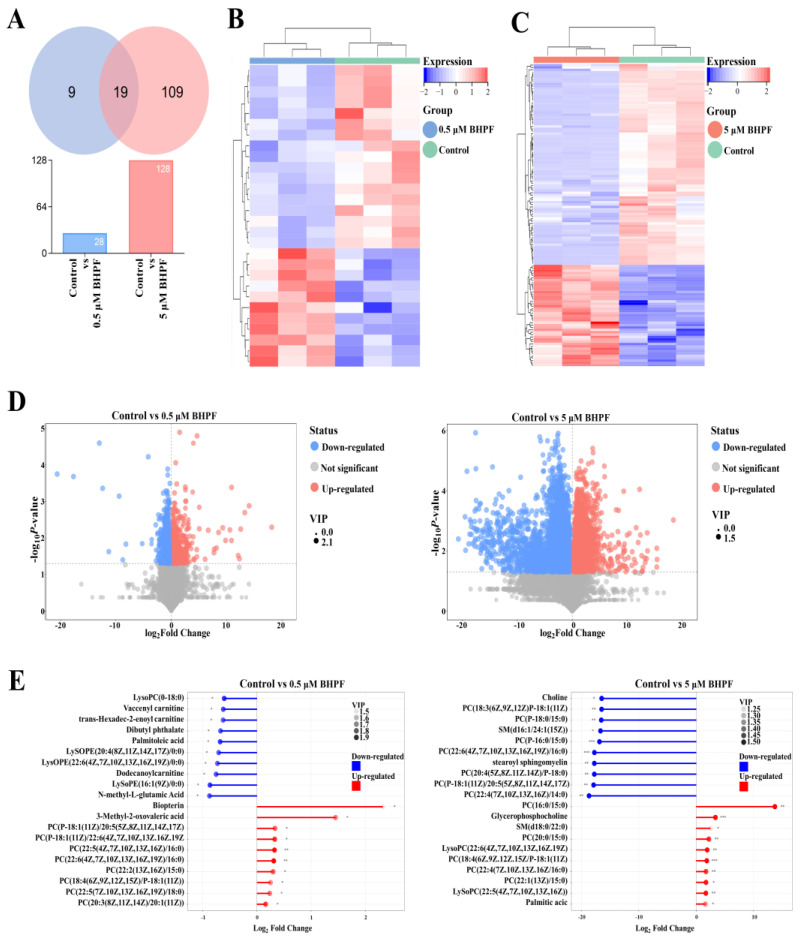
Metabolomics profiles of Ishikawa cells exposed to BHPF. (**A**) Numbers of up- and downregulated DEMs in different BHPF-treatment groups. (**B**,**C**) Heatmap of hierarchical clustering analysis for DEMs between different groups. (**D**) Volcano plot of BHPF-induced DEMs in the control vs. 0.5 μM and 5 μM groups. (**E**) Up- and downregulation of the top 20 DEMs. Each group consisted of *n* = 3, * *p* < 0.05, ** *p* < 0.01, *** *p* < 0.001.

**Figure 3:**
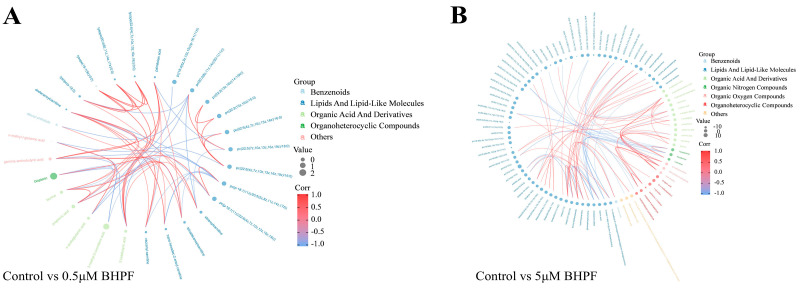
Chord plot analysis of interrelation of DEMs in the two BHPF-treatment groups. (**A**) Chord plot analysis of interrelation of DEMs between Control and low-exposure group. (**B**) Chord plot analysis of interrelation of DEMs between Control and high-exposure group.

We identified 28 and 128 DEMs in the 0.5 μM and 5 μM BHPF exposure group, respectively, compared to the control, and the Venn diagram displays the number of common DEMs identified at different exposure concentrations of BHPF (Figure 2A and Table S3). More DEMs were detected at 5 μM than at 0.5 μM compared to the control. Hierarchical clustering analysis was conducted, and a heatmap for each comparison group is shown (Figure 2B,C). Volcano plots were generated to illustrate the distribution of DEMs among the comparison groups (Figure 2D). The top 20 significant DEMs in each comparison group are presented in Figure 2E. Moreover, we analyzed the correlations among the DEMs and visualized the relationships via chord plots (Figure 3). In the chord plots, metabolite classes are arranged around the circle, with a total of seven distinct classes represented.

#### 3.2.3. KEGG Pathway Enrichment Analysis

**Figure 4:**
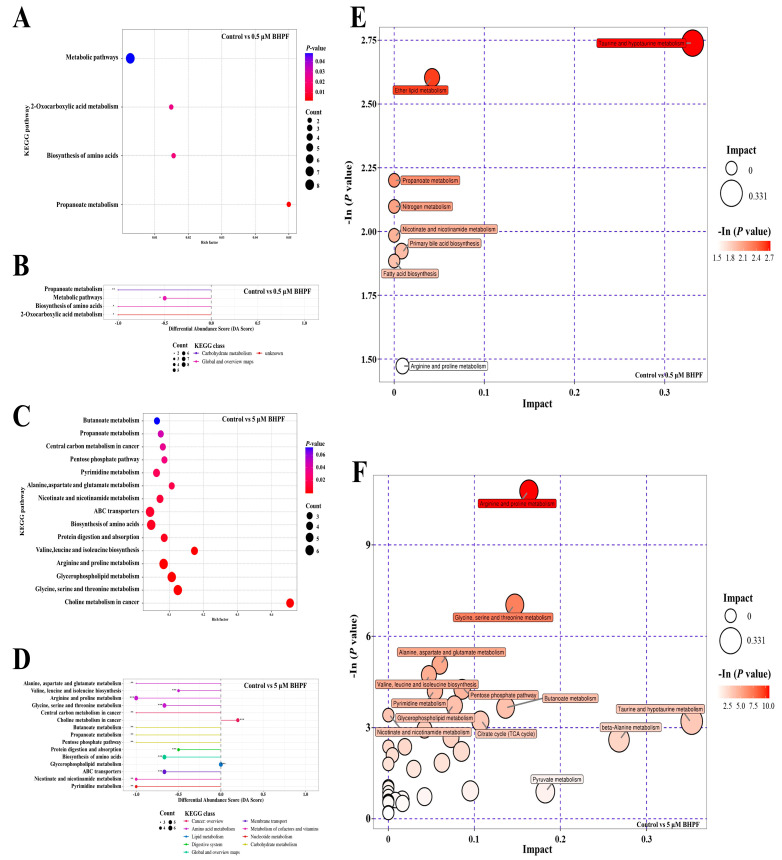
Enrichment analysis of DEMs. (**A**,**C**) KEGG enrichment based on the rich factor for DEMs in the control vs. 0.5 μM and 5 μM BHPF exposure groups. (**B**,**D**) Differential abundance score for DEMs. (**E**,**F**) Pathway analysis for DEMs in different groups. * *p* < 0.05, ** *p* < 0.01, *** *p* < 0.001.

To identify specific metabolic pathways altered by different concentrations of BHPF in endometrial epithelial cells, KEGG pathway analysis of the DEMs was performed. The results revealed that the DEMs in the 0.5 μM BHPF exposure group were considerably enriched in propanoate metabolism, the biosynthesis of amino acids, and 2-oxocarboxylic acid metabolism (Figure 4A,B). Moreover, 44 metabolic pathways were identified in the 5 μM BHPF exposure group (Table S4), including choline metabolism in cancer, arginine and proline metabolism, glycine, serine, and threonine metabolism, the biosynthesis of amino acids, and ABC transporters. Fifteen pathways are presented in Figure 4C,D.

Moreover, to identify the pathways that were most closely associated with DEMs, MetaboAnalyst was applied for metabolic pathway enrichment via topological analysis. A total of 8 and 36 metabolic pathways were detected in the 0.5 μM and 5 μM BHPF exposure groups, respectively (Figure 4E,F). Taurine and hypotaurine metabolism, ether lipid metabolism, arginine and proline metabolism, and primary bile acid biosynthesis were the major four significantly different pathways between the 0.5 μM BHPF exposure group and the control group (Figure 4E). In addition, these four pathways were likewise regulated in the 5 μM BHPF exposure group compared with the control group. These findings suggest that the commonly regulated pathways may play a crucial role in the pathogenic mechanisms of BHPF after exposure. Furthermore, uniquely regulated pathways, such as beta-alanine metabolism, sphingolipid metabolism, D-glutamine and D-glutamate metabolism, the citrate cycle, and butanoate metabolism, could be involved in specific pathways when exposed to 5 μM BHPF (Figure 4F).

### 3.3. Transcriptomic Analysis

#### 3.3.1. Transcriptome Sequencing and DEG Identification

To investigate the molecular impact of BHPF on the endometrial epithelial cells, gene expression profiles of the control and BHPF exposure groups were constructed via transcriptomic analysis. Nine samples yielded 52.95 Gb of clean data in total. Table S5 provides a full summary of detailed information on the raw reads, clean reads, and Q20 and Q30 values, all of which suggest the high reliability of the sequencing data. The mapping efficiency of these samples with the reference genome was greater than 96% (Figure S3).

**Figure 5:**
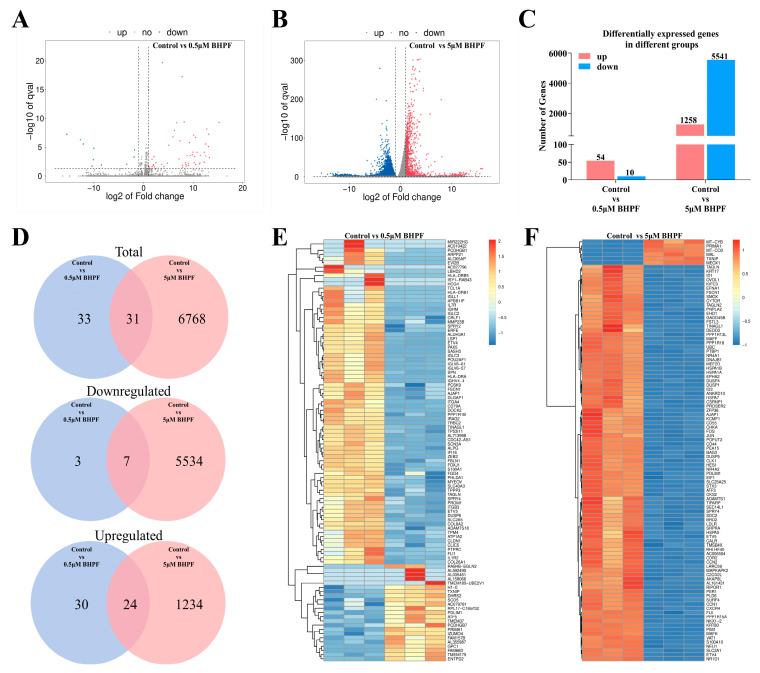
Transcriptomic analysis in Ishikawa cells after BHPF exposure. (**A**) Volcano plot illustrating the DEGs identified in the transcriptomes of the control vs. 0.5 μM BHPF exposure group. (**B**) Volcano plot of DEGs of the control vs. 5 μM BHPF exposure group. (**C**) Number of upregulated and downregulated DEGs in different comparison groups. (**D**) Venn diagram showing the significantly DEGs in each pairwise comparison and the overlap among them. (**E**,**F**) Heatmap of DEGs between the different comparison groups. Each group consisted of *n* = 3.

Differential expression analysis of endometrial cells exposed to two concentrations of BHPF was performed, and the DEGs identified in this study presented a minimum two-fold change (absolute log2 fold change with FPKM ≥ 1) and significant difference (false discovery rate (FDR) < 0.05). Volcano plots were created to visualize the differential gene distribution between cells exposed to BHPF and cells treated with 0.02% DMSO (control). The blue and red dots in Figure 5A,B represent the significantly downregulated and upregulated genes, respectively. A total of 64 DEGs (54 upregulated and 10 downregulated) were detected between the 0.5 μM BHPF exposure group and the control group, whereas 6799 DEGs (1258 upregulated and 5541 downregulated) were detected between the 5 μM BHPF exposure group and the control group. More genes were upregulated in the 0.5 μM BHPF exposure group, whereas more genes were downregulated in the 5 μM BHPF exposure group (Figure 5C). Additionally, we compared all the aforementioned DEGs and identified certain DEGs regulated by different BHPF concentrations (Figure 5D). The 5 μM BHPF exposure group clearly presented more specific DEGs than those in the 0.5 μM BHPF exposure group, indicating a difference between 5 μM and 0.5 μM BHPF exposure at the transcriptomic level. To further illustrate the differences between the DEGs in the three groups, heatmaps were created in accordance with the top 100 DEGs with the lowest q values (Figure 5E,F).

#### 3.3.2. GO and KEGG Enrichment Analysis

**Figure 6:**
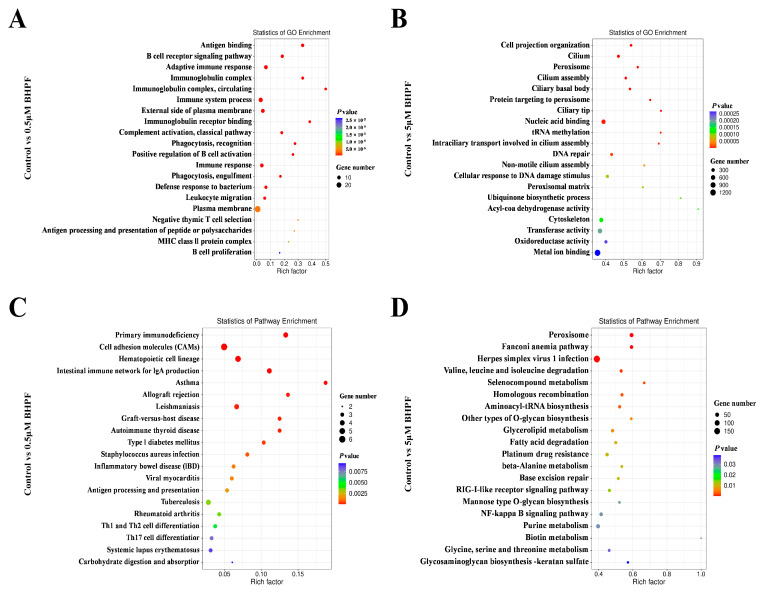
Enrichment analysis of DEGs. (**A**,**B**) Scatter plot of the top 20 significantly enriched GO terms. (**C**,**D**) KEGG pathway enrichment of DEGs from different comparison groups.

To explore the response of endometrial cells to BHPF further, GO enrichment analysis was used to functionally annotate the DEGs. GO annotation included biological process (BP), molecular function (MF), and cellular compartment (CC) terms (Figure S4A,B), and the top 20 significantly enriched GO terms are shown in Figure 6A,B. In the 0.5 μM BHPF exposure group, the most significant GO terms were enriched in antigen binding, the B-cell receptor signaling pathway, the adaptive immune response, and the immunoglobulin complex, which are associated mainly with the immune response (Figure 6A). However, in the 5 μM BHPF exposure group, DEGs were significantly enriched in signal transduction, ion transport, DNA repair, cytoskeleton, and oxidation–reduction processes (Figure 6B). These results indicate that a low concentration of BHPF activates the immunological response of endometrial cells and that an increased concentration of BHPF exposure influences cell signal transduction and other biological processes, resulting in a reduction in cell viability. This finding is consistent with the inhibitory effect on cell viability caused by high BHPF concentrations (Figure 1B).

Moreover, a KEGG pathway enrichment analysis of the DEGs was performed to illustrate the pivotal pathway changes in endometrial cells exposed to different concentrations of BHPF. Compared with those in the control group, the pathways enriched in the 0.5 μM BHPF exposure group were associated with primary immunodeficiency and cell adhesion molecules (Figure 6C). For 5 μM BHPF exposure, the majority of the DEGs were related to amino acid metabolism, lipid metabolism, the RLR signaling pathway, and the NF-κB signaling pathway (Figure 6D).

### 3.4. Joint Analysis of Metabolome and Transcriptome

Furthermore, an integrative analysis was performed, correlating the transcriptome with the metabolome by examine the relationships between DEGs and DEMs. According to the shared pathways by KEGG analysis in transcriptomics and metabolomics (Table S6), the abundance of DEMs and DEGs involved in these pathways was analyzed, and the Pearson correlation coefficients are shown in Table S7.

**Figure 7:**
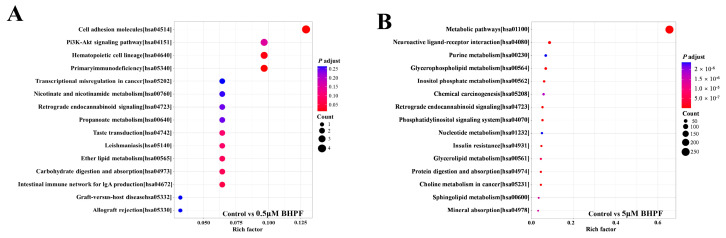
Joint analyses of differential transcripts and metabolites. (**A**) Bubble plot of joint KEGG enrichment analysis between the DEMs and DEGs in the control vs. 0.5 μM BHPF exposure group. (**B**) Bubble plot joint KEGG enrichment analysis between the DEMs and DEGs in the control vs. 5 μM BHPF exposure group.

We then performed a joint KEGG enrichment analysis between the DEMs and DEGs to identify key metabolites and functional genes implicated in BHPF exposure in endometrial cells. Two pathways, namely, primary immunodeficiency [hsa05340] and cell adhesion molecules [hsa04514], were significantly enriched in the 0.5 μM BHPF exposure group (Figure 7A and Table S8). Therefore, the DEMs and DEGs involved in these two pathways were integrated and analyzed. Taking the primary immunodeficiency as an example, genes encoding *IL7R* and *PTPRC* were upregulated in endometrial cells under 0.5 μM BHPF exposure; moreover, DEMs such as propionic acid, taurine, dodecanoylcarnitine, trans-Hexadec-2-enoyl carnitine, and tetradecanoylcarnitine were enriched in the related metabolic pathways. As BHPF accumulated at a relatively high concentration, the number of signaling pathways affected in endometrial cells increased dramatically (Figure 7B and Table S8). We observed that 81 pathways were significantly enriched when the cells were exposed to 5 μM BHPF. The affected pathways play important roles in amino acid metabolism, energy metabolism, and nucleotide metabolism, and include the crucial signal transduction pathways, such as the PI3K-Akt signaling pathway, the AMPK signaling pathway, and the TNF signaling pathway, etc. Therefore, we propose that the metabolites and genes associated with these signaling pathways play pivotal roles in the functional abnormalities of endometrial cells caused by the cytotoxicity of high BHPF concentrations.

## 4. Discussion

In this study, we pioneered an exploration into the effects and underlying mechanisms of BHPF exposure on the endometrial epithelial functions by in vitro experiments and integrative metabolomics with transcriptomics analyses. We demonstrated that BHPF exerts cytotoxic effects on endometrial epithelial cells, including alterations in cell viability, metabolic changes, and aberrant gene expression at the transcriptional level, which suggests potential health risks of BHPF.

Metabolomics revealed 28 DEMs after exposure to 0.5 μM BHPF, whereas the number of DEMs increased significantly to 128 after exposure to 5 μM BHPF. Pathway analysis revealed that four pathways were significantly affected in the two groups, namely taurine and hypotaurine metabolism, ether lipid metabolism, arginine and proline metabolism, and primary bile acid biosynthesis. We found that the taurine and hypotaurine metabolism pathways and the primary bile acid biosynthesis pathway exert biological effects through a reduction in taurine levels. As reported, taurine can protect cells from oxidative stress damage and regulate anti-inflammatory responses to maintain the immune homeostasis, and is ubiquitous in the uterus [24,25]. Our data demonstrated a reduction in taurine levels in both the low-concentration group (0.5 μM BHPF) and the high-concentration group (5 μM BHPF), suggesting that endometrial epithelial cells may be more susceptible to oxidative damage, leading to an imbalance in the immune inflammatory response and increases in the susceptibility of the immune system to inflammation caused by exposure to BHPF. Moreover, ether lipid metabolism, along with arginine and proline metabolism, has been proven to have significant associations with immune system, tumor growth, and invasion, among other processes [26,27,28,29]. These pathways play essential roles in various biological processes, including signaling transduction, the immune response, cell growth, and so on, highlighting that endometrial epithelial cells trigger immunological reactions persistently, activate important signaling pathways, and ultimately impact cell viability when cells are exposed to BHPF at low to high concentrations. Furthermore, the 5 μM BHPF exposure group presented unique regulated pathways, primarily associated with energy metabolism and amino acid metabolism. We mainly found that pyruvic acid decreased significantly by approximately 3.6 times in the 5 μM BHPF exposure group and that beta-alanine decreased by more than 6.7 times. Pyruvic acid is well known to participate in pyruvate metabolism, butanoate metabolism, the citrate cycle, alanine, aspartate, and glutamate metabolism. Reduction of pyruvic acid impairs the synthesis of ATP, amino acids, lipids, and proteins; and influence cellular processes, resulting in the disruption of the cell proliferation, differentiation, and survival of endometrial cells [30,31,32]. In addition, beta-alanine is a crucial nonessential amino acid that participates in the synthesis of important coenzymes, such as coenzyme A (CoA), influencing cellular metabolism and energy utilization [33,34]. Additionally, it can neutralize intracellular free radicals, mitigate oxidative stress-induced cellular damage, and maintain cellular homeostasis [35]. Thus, the high-concentration exposure of BHPF to endometrial epithelial cells results in a decreased level of beta-alanine, thereby leading to disrupted cellular energy metabolism and ultimately causing severe adverse effects on cellular function. To explore the underlying molecular mechanism by which BHPF exerts adverse effects on endometrial epithelial cells, a transcriptome analysis was performed. Transcriptomic analysis revealed that primary immunodeficiency was the main enriched biological process after low-concentration exposure to BHPF. With increasing exposure concentration, multiple signaling pathways related to metabolism, the immune response, DNA damage, and oxidation–reduction process, such as the RLR signaling pathway and the NF-κB signaling pathway, were affected, which is consistent with the metabolomics analysis results.

By combining the data obtained from untargeted metabolomics and transcriptomics, we observed a significant upregulation of *IL7R* and *PTPRC* in the 0.5 μM BHPF exposure group, and validated the expression level by qRT-PCR (Figure S5). Research has indicated that *IL7R* is upregulated and enhances responsiveness to IL-7 signaling pathway to augment the effectiveness of immune responses when subjected to immune stimulation [36,37]. Moreover, CD45, encoded by *PTPRC,* is a crucial phosphatase located on the cell membrane. Upon the external stimuli-induced upregulation of *PTPRC*, CD45 modulates the phosphorylation levels of receptors and signaling molecules, thereby influencing cell survival and proliferation and participating in cellular immune responses [38,39]. Therefore, we found that exposure to low concentrations of BHPF in endometrial epithelial cells activated receptors and signaling pathways associated with primary immunodeficiency, such as *IL7R* and *PTPRC*, which may trigger inflammatory responses and sustain cell survival through the continuous consumption of taurine. For the 5 μM BHPF exposure group, our findings indicate a significant enrichment of metabolic signaling pathways, particularly the purine metabolism pathway. The investigation revealed a notable upregulation of *PGM1*, alongside a significant downregulation of the genes *PDE3B*, *ENTPD8,* and *AK9* within the purine metabolism pathway (Figure S5). The literature corroborates the critical role of the *ENTPD8* gene in mediating intracellular purine and pyrimidine metabolism [40]. The downregulation of *ENTPD8* potentially disrupts DNA and RNA synthesis and repair mechanisms, consequently inducing metabolic dysregulation. Furthermore, aberrant conversion of NDP and NMP catalyzed by *ENTPD8* profoundly impacts cellular energy generation and utilization [41,42,43]. Moreover, it is well known that *PGM1*, *PDE3B*, and *AK9* are crucial regulatory genes in cellular energy metabolism. Studies have shown that *PGM1* encodes phosphoglucomutase 1, which participates in the regulation of both gluconeogenesis and glycolysis pathways, maintaining cellular energy balance and influencing cell viability and proliferation [44,45]. *PDE3B*, as a member of the phosphodiesterase family, modulates intracellular signal transduction and is involved in the regulation of cell growth and energy homeostasis [46,47]. Additionally, *AK9* is closely associated with intracellular energy metabolism and nucleic acid synthesis, and may be involved in the cell’s response to energy depletion and stress, regulating the adaptive mechanisms of the cell [48]. Consequently, the upregulation of *PGM1* expression, coupled with the downregulation of *PDE3B* and *AK9*, may have a significant impact on cellular energy metabolism, signal transduction, and processes such as cell growth and proliferation. Therefore, our functional experiments demonstrated that the exposure of endometrial epithelial cells to high concentrations of BHPF significantly inhibited cell viability. The underlying molecular mechanism involves the aberrant expression of key genes in the purine metabolism pathway, triggering stress responses that disrupt cellular metabolism and ultimately lead to a significant reduction in cell viability.

Nonetheless, the empirical results reported here should be considered in the light of several limitations. One concern with this study is that although Ishikawa cells are a standard cell model for research purposes, the consideration of cellular heterogeneity along with the distinctions between in vitro and in vivo experiments suggests that future investigations using animal models or human tissues should be performed for more profound exploration. Second, this study represents a short-term acute investigation in which we explored the effects of acute exposure to low-concentration BHPF in endometrial epithelial cells. Subsequent studies may consider extending the duration of exposure, whether in vivo or in vitro. Additionally, the multiomics data from this study demonstrate that the exposure of endometrial epithelial cells to BHPF significantly affects cell adhesion, ion transport, and other associated functions, which provide new insights for future research. Building on these findings, future studies will aim to investigate the regulatory mechanisms informed by the omics analysis results, to further elucidate the underlying molecular mechanisms.

## 5. Conclusions

Through comprehensive multiomics studies, we elucidated the toxic effects of BHPF exposure on endometrial epithelial cells. Specifically, at low concentrations, cells do not undergo changes in cell viability or phenotype; however, immune stress and inflammatory responses, accompanied by significant alterations in metabolite levels and metabolic dysregulation, were still evident in the cells. As BHPF gradually accumulates at high concentrations, numerous signaling pathways in cells undergo substantial changes, accompanied by abnormal gene expression levels and significant alterations in the metabolic profile, thus leading to a significant impairment in cell viability.

## Data Availability

The data presented in this study are available on request from the corresponding author.

## Supplementary Materials

The following supporting information can be downloaded at: https://www.mdpi.com/article/10.3390/toxics13020100/s1, Figure S1: Non-targeted metabolomic analysis of Ishikawa cells under BHPF treatment; Figure S2: PCA and OPLS -DA score plot of metabolites in samples; Figure S3: Distribution of reference genome alignment regions of all samples; Figure S4: GO enrichment analysis histogram of DEGs; Figure S5: Indicated genes were validated by qRT-PCR in 0.5 μM BHPF exposure cells (A) and 5 μM BHPF exposure cells (B) compared to the control group; Table S1: K-Means Analysis data matrix; Table S2: Venn matrix; Table S3: KEGG pathway enrichment analysis of DEMs in different concentrations of BHPF exposure; Table S4: Mapping efficiency of all samples with the reference genome; Table S5: The shared pathways by KEGG analysis in transcriptomics and metabolomics; Table S6: pathways by KEGG analysis in transcriptomics and metabolomics; Table S7: Joint KEGG enrichment analysis between the DEMs and DEGs after BHPF exposure; Table S8: The primers for qPCR.
