# Supplementary material for: Integrated Metabolomic and Transcriptomic Analysis Revealed the Mechanism of BHPF Exposure in Endometrium
Source: Toxics. 2025 Jan 27;13(2):100. doi: 10.3390/toxics13020100 (PMC11861605; doi:10.3390/toxics13020100)
Supplement: Supplementary file 1 [file toxics-13-00100-s001.zip › Supplementary Figures-toxics.pdf]

Figure S1

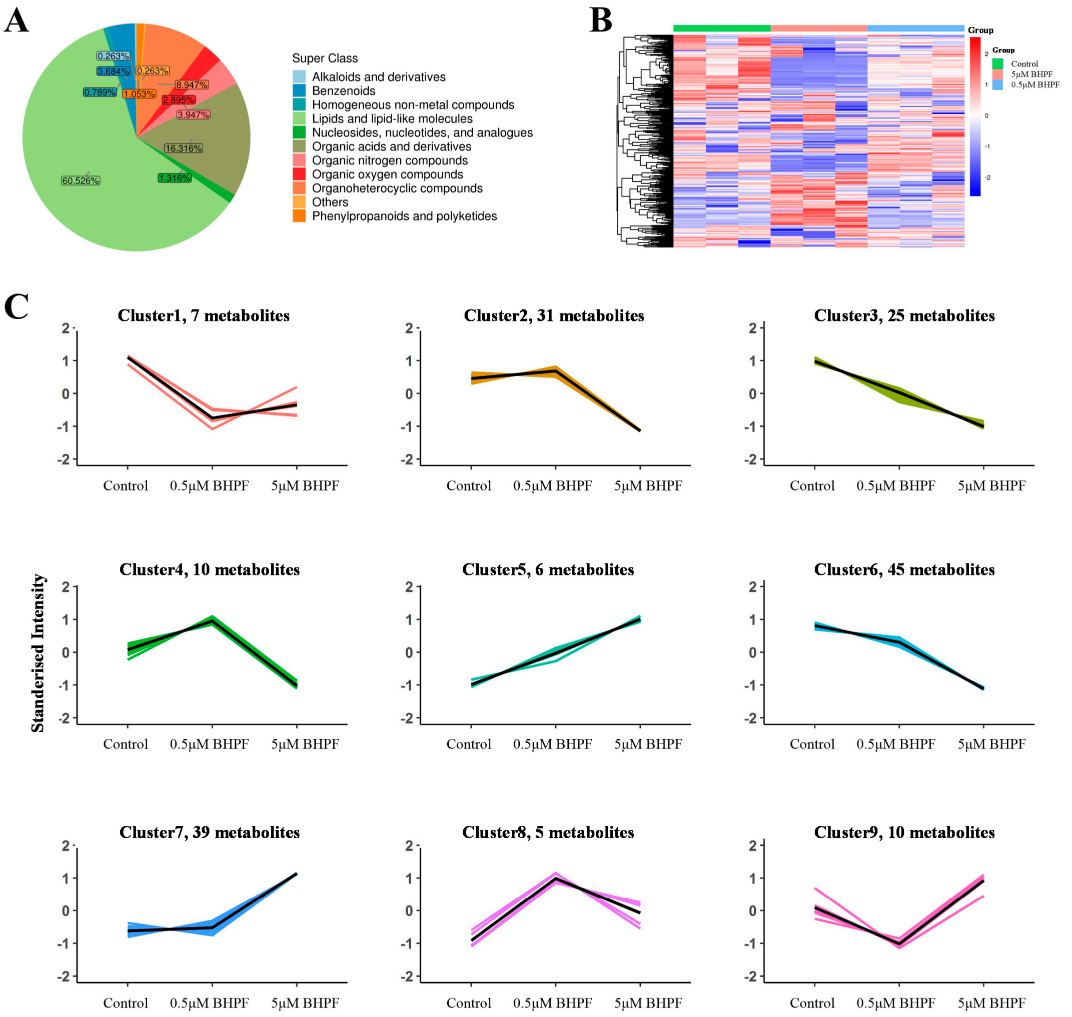

Figure S1. Non-targeted metabolomic analysis of Ishikawa cells under BHPF treatment. (A) Pie plot of the annotated metabolites classification. (B) Heatmap of hierarchical clustering analysis for all groups. (C) K-means cluster analysis of the differentially accumulated metabolites.

Figure S2

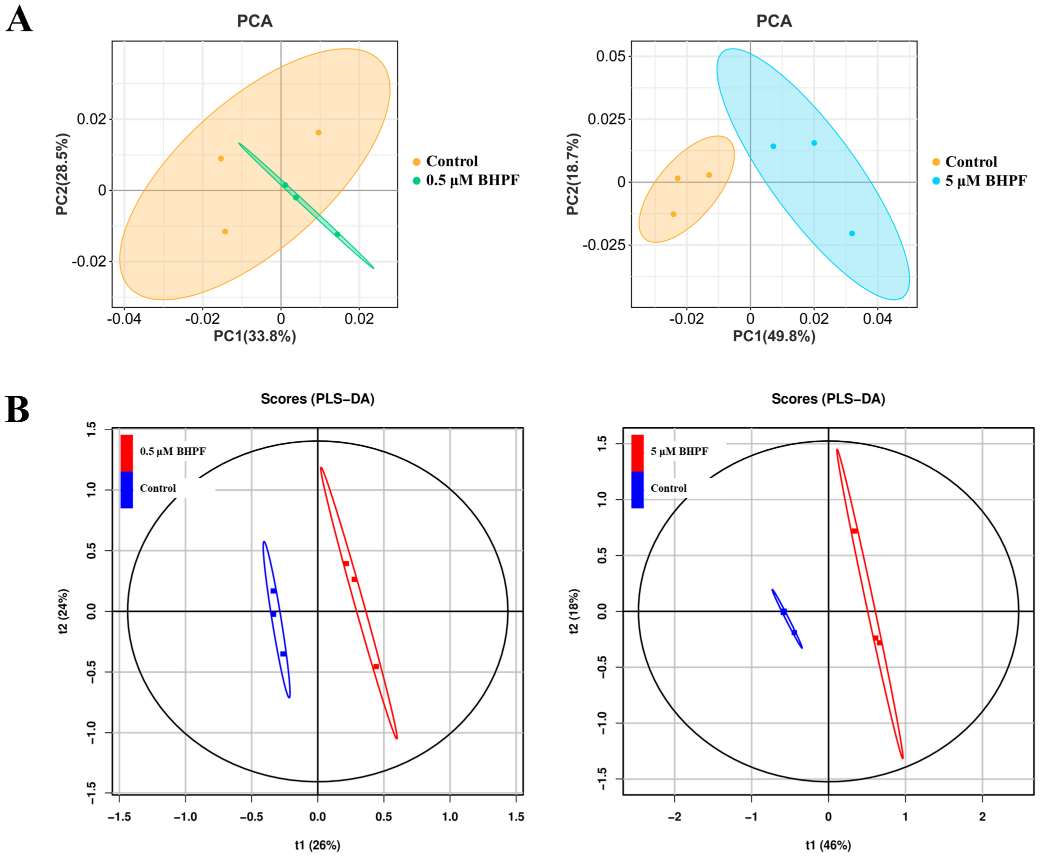

Figure S2. PCA and OPLS -DA score plot of metabolites in samples. (A, B) Score Scatter plot of PCA model. (C, D) Score scatter plot of OPLS-DA model.

Figure S3

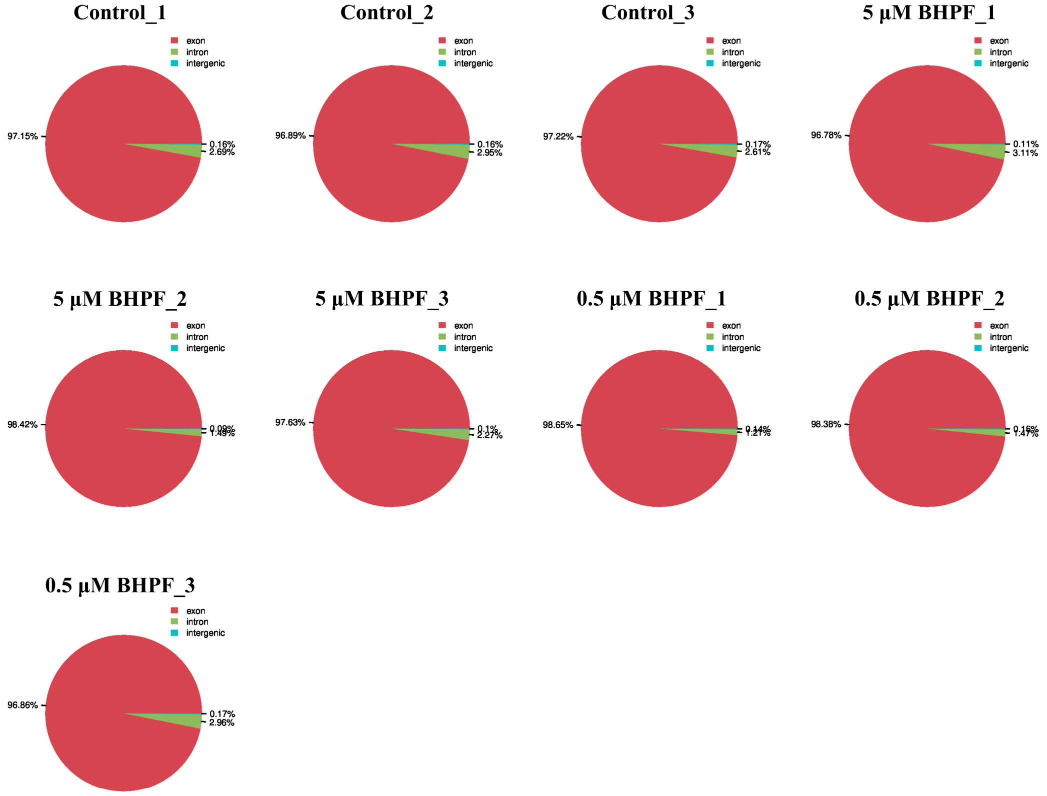

Figure S3. Distribution of reference genome alignment regions of all samples.

Figure S4

A

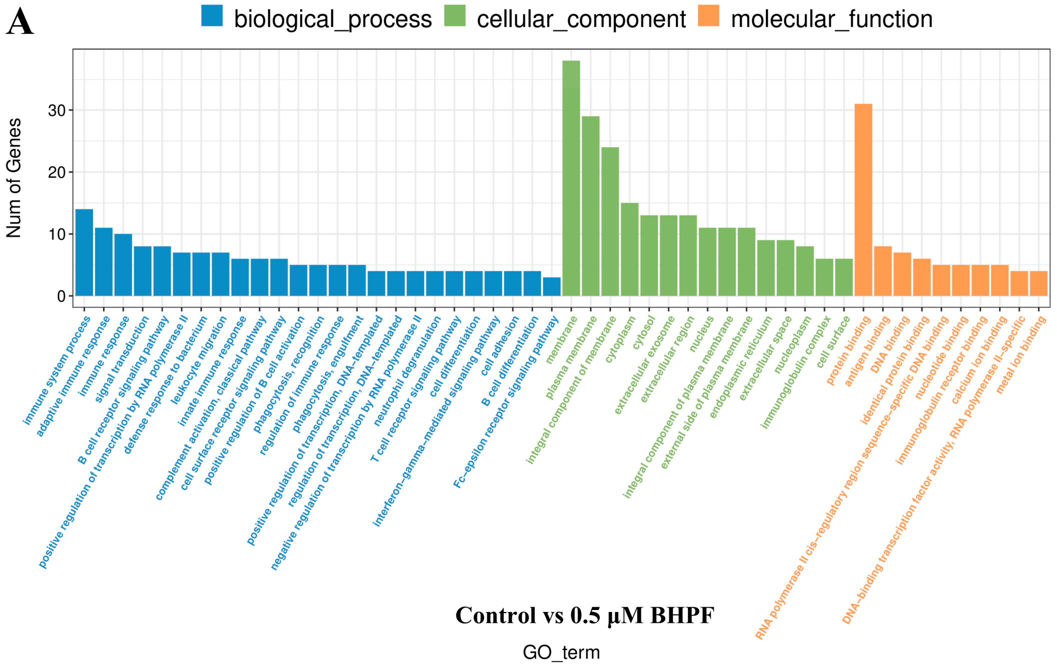

B

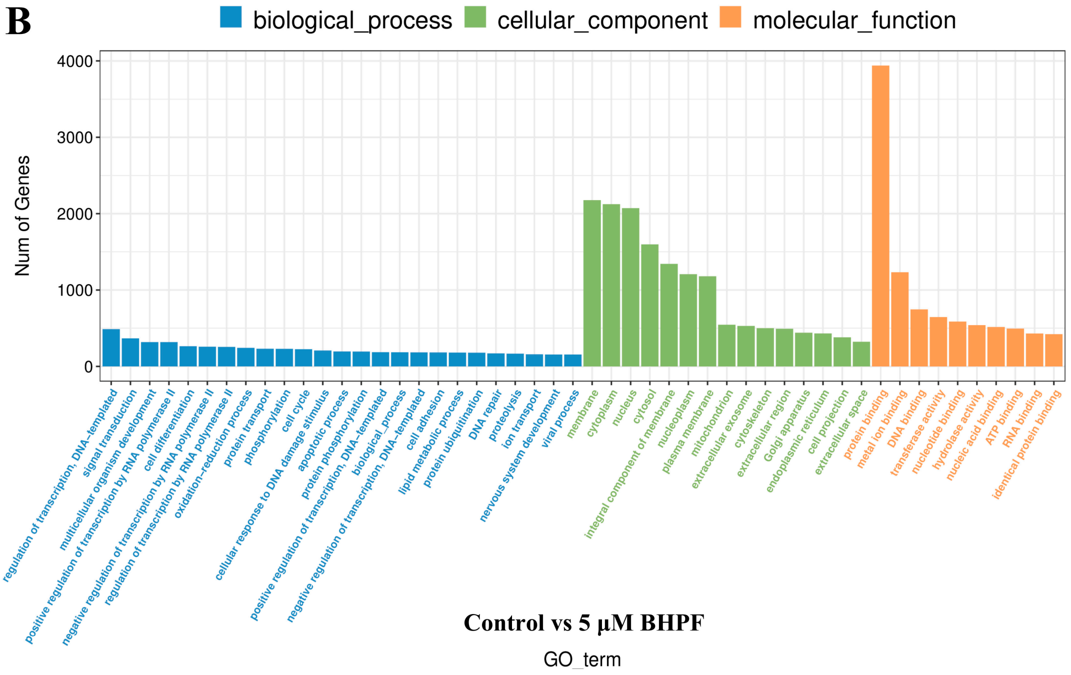

Figure S4. GO enrichment analysis histogram of DEGs detected in Control vs 0.5 μM BHPF exposure group (A) and Control vs 5 μM BHPF exposure group.

Figure S5

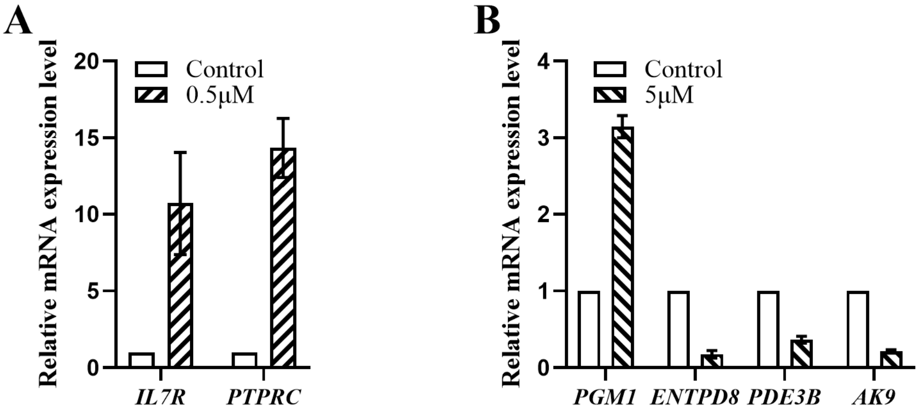

Figure S5. Indicated genes were validated by qRT-PCR in 0.5 μM BHPF exposure cells (A) and 5 μM BHPF exposure cells (B) compared to the control group.
